# The Complex Mechanisms by Which Neurons Die Following DNA Damage in Neurodegenerative Diseases

**DOI:** 10.3390/ijms23052484

**Published:** 2022-02-24

**Authors:** Sina Shadfar, Mariana Brocardo, Julie D. Atkin

**Affiliations:** 1 Centre for Motor Neuron Disease Research, Macquarie Medical School, Macquarie University, Sydney, NSW 2109, Australia; sina.shadfar@mq.edu.au (S.S.); mariana.brocardo@mq.edu.au (M.B.); 2 Department of Biochemistry and Genetics, La Trobe Institute for Molecular Science, La Trobe University, Bundoora, Melbourne, VIC 3086, Australia

**Keywords:** DNA damage, cell death mechanisms, neurodegenerative disease

## Abstract

Human cells are exposed to numerous exogenous and endogenous insults every day. Unlike other molecules, DNA cannot be replaced by resynthesis, hence damage to DNA can have major consequences for the cell. The DNA damage response contains overlapping signalling networks that repair DNA and hence maintain genomic integrity, and aberrant DNA damage responses are increasingly described in neurodegenerative diseases. Furthermore, DNA repair declines during aging, which is the biggest risk factor for these conditions. If unrepaired, the accumulation of DNA damage results in death to eliminate cells with defective genomes. This is particularly important for postmitotic neurons because they have a limited capacity to proliferate, thus they must be maintained for life. Neuronal death is thus an important process in neurodegenerative disorders. In addition, the inability of neurons to divide renders them susceptible to senescence or re-entry to the cell cycle. The field of cell death has expanded significantly in recent years, and many new mechanisms have been described in various cell types, including neurons. Several of these mechanisms are linked to DNA damage. In this review, we provide an overview of the cell death pathways induced by DNA damage that are relevant to neurons and discuss the possible involvement of these mechanisms in neurodegenerative conditions.

## 1. Introduction

DNA is essential for life, but it is highly reactive and thus susceptible to ongoing damage. The DNA damage response (DDR) is therefore continuously activated to counteract the effects of lesions to DNA. This involves cellular mechanisms to both detect and repair DNA damage. Importantly, the DDR can differentiate between DNA damage that is either repairable or unrepairable. Hence, efficient DNA repair processes are essential for cellular viability, although they decline significantly during aging. DNA damage and defective DNA repair mechanisms are now increasingly implicated in age-related neurodegenerative diseases as drivers of the disease process.

The DDR is initially protective, but ironically, if DNA lesions cannot be repaired, the DDR induces cell death to protect the genome and hence prevent the incorporation of mutations. Indeed, induction of DNA damage underlies many chemotherapy approaches to selectively trigger the death of tumor cells. There are many different mechanisms by which a cell can die, and our understanding of these processes has increased significantly in recent years. However, whilst cell death mechanisms are available to all cell types, they are selectively induced and in most mature cells, death is normally suppressed. However, neurons are post-mitotic and thus need to be maintained for the lifespan of the organism. Hence, induction of cell death in neurons is potentially more deleterious than in other cell types. Efficient DNA repair processes are therefore important to maintain the survival of neurons in response to normal aging and environmental insults. Several cell death mechanisms are known to be induced following DNA damage, and important DDR-related cell death proteins, particularly p53, are increasingly linked to neurodegenerative diseases. This review will discuss our current knowledge of the mechanisms of cell death induced by DNA damage in neurons and their potential importance in neurodegenerative disorders.

## 2. The DNA Damage Response

DNA damage is defined as any modification of DNA that changes its coding properties or normal function in transcription or replication [1]. Genomes are modified by reactive molecules that are produced endogenously or by environmental/exogenous physical, chemical, and biological agents. These include ultraviolet (UV) light, ionizing radiation (IR), heavy metals, air pollutants, chemotherapeutic drugs, and inflammatory responses [2,3]. There are various types of DNA damage. They can involve chemical modification of a base, including methylation, alkylation, oxidation, or loss of a base. Alternatively, DNA replication errors can occur, or a break in the phosphodiester bonds can result in either one (single-stranded breaks, SSBs) or both DNA strands (double-stranded breaks, DSBs) [4,5]. SSBs are much more common than DSBs, but the latter are much more deleterious, and SSBs can also convert to DSBs [6].

To deal with the presence of DNA damage, cells activate the DDR. This is a complex and co-ordinated cellular signalling network that senses, signals, repairs, and thus responds to damaged DNA. After sensing genotoxic insults, the DDR amplifies and propagates these signals (which can involve rapid post-translational protein modifications) and recruits various proteins to the broken DNA ends and surrounding chromatin [7]. Depending on the duration and severity of DNA damage, the cell can initially choose to repair the lesion as a pro-survival mechanism. However, if the damage cannot be repaired, the cell may enter either permanent cell-cycle arrest or a regulated program of death, to protective the organism from cells with a defective genome [7]. These signalling mechanisms therefore represent a cascade of events designed to preserve genome integrity.

### 2.1. DNA Damage Signalling in Neurons

To date, the DNA damage response has been investigated primarily in proliferating cells, where it is closely associated with the cell-cycle machinery. However, neurons lack cell-cycle checkpoints. Therefore, they require different strategies to deal with the accumulation of DNA lesions, and thus maintain genome integrity, than proliferating cells. Furthermore, neurons are also particularly vulnerable to DNA damage, given their post-mitotic nature. However, in contrast to proliferating cells, in neurons the DDR has not been well characterised, and it appears to be limited to active genes [8]. In the sections below, we will discuss the DDR, with a particular focus on the processes relevant to neurons, rather than those present in proliferating cells and other cell types. 

### 2.2. Detecting the Presence of DNA Damage

**Figure 1:**
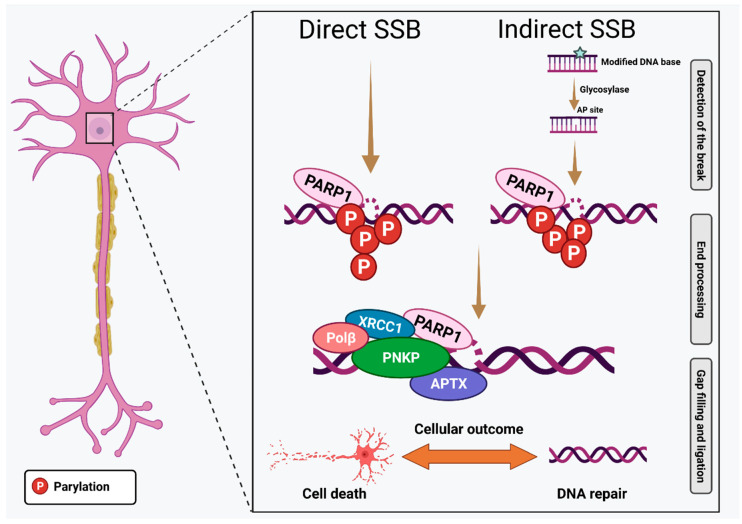
A simplified diagram illustrating the recognition of DNA single-strand breaks (SSBs) and the involvement of PARP1 in sensing the breaks. In neurons, SSBs can occur either by reactive oxygen species (ROS) that damage the deoxyribose sugar backbone (direct SSB), or by the action of enzymes that recognise and remove the modified DNA bases by oxidation, deamination, alkylation, and hydrolysis (indirect SSB). Excision of the damaged base is followed by PARP1 binding which then acts on itself, resulting in the addition of long branching chains of poly (ADP-ribose) (parylation). After detection of the DNA damage, X-ray repair cross complementing protein 1 (XRCC1) recruits several key proteins needed for broken DNA end processing and DNA ligation. This includes DNA polymerase β (POLβ), polynucleotide kinase phosphatase (PNKP), and aprataxin (APTX), which resolve the abnormal 5′ or 3′ ends, facilitating DNA repair, gap filling, and nick ligation. Hence, PARP1 plays an important role in the detection of SSBs [10,19].

Whilst SSBs are not as genotoxic as DSBs, they can arise within the cell in large numbers, and are deleterious if left unrepaired. In addition, SSBs can rapidly convert to DSBs [9]. SSB lesions are particularly relevant to neurons due to the rich oxygen environment of the brain, and they can arise from many sources. Hence, neurons are exposed to particularly high levels of SSBs during their long life. Interestingly, a recent study demonstrated that neurons accumulate unexpectedly high levels of SSBs at specific sites within the genome, the epigenetic machinery of active enhancers [8]. SSB repair at localised regions implies that DNA may be continuously damaged in neurons, but a rapid and efficient response to SSB formation is required for continued neuronal viability [10]. Repair of SSBs is facilitated by the ADP-ribosylating enzyme poli-(ADP-ribose) polymerase (PARP1), which is essential for initiating various types of DNA repair. PARP1 transfers successive units of ADP-ribose (PAR) onto residues of target proteins, including itself [11,12]. Normally, activation of PARP1 facilitates DNA repair and survival in response to mild genotoxic stress [13,14]. However, persistent activation of PARP1 is a feature of several pathological conditions, including neurodegenerative diseases [15,16], inflammatory disorders [17], and cancer. This leads to overconsumption and thus a reduction in the levels of NAD^+^, resulting in energetic/mitochondrial dysregulation [18] (Figure 1).

DSBs are sensed by two protein complexes, Ku70/80 and MRN. Ku is a heterodimer composed of two polypeptides, Ku70 (XRCC6) and Ku80 (XRCC5). It binds to the ends of DNA, creating a ring-like structure near the break site [20]. This allows binding of the catalytic subunit of the DNA-dependent serine/threonine protein kinase (DNA-PKcs) to create an activated complex that signals, recruits, and activates downstream nucleases, polymerases, and ligases [21]. DNA-PKcs is further phosphorylated by the kinase ataxia–telangiectasia mutated (ATM) [22]. 

The MRN complex contains MRE11, RAD50, and NBS1 (MRN) proteins. It recognizes and binds to the two ends of the DSB to mediate ATM and DNA-PK DNA damage responses [23,24]. To amplify local signalling at sites of DNA damage, the H2A histone family member X (H2AX) is phosphorylated by activated ATM in cis on Ser139 to form γH2AX [25]. This leads to recruitment of mediator of DNA damage checkpoint protein 1 (MDC1) and chromatin remodelling proteins p400 and Tip60, which modify the structure of chromatin to allow the entry of other proteins [26,27]. This includes p53 binding protein 1 (53BP1), mediator of DNA damage checkpoint protein 1 (MDC1)*,* and breast cancer gene 1 (BRCA1), which activates ATM [28,29,30]. This recruitment establishes an effective positive feedback loop that leads to the formation of cytologically detectable foci, which are a widely used marker of DNA damage [31]. Ubiquitin signaling plays an important role in coordinating the recruitment of DNA repair factors such as BRCA1 and 53BP1. Two critical proteins in this early DNA damage signaling event are the RING-type ubiquitin E3 ligases RNF8 and RNF168. MDC1 engages RNF8, which subsequently facilitates recruitment of RNF168. RNF168 then promotes the ubiquitination of histone H2A/H2AX, which is important for the recruitment of 53BP1 and BRCA1 [31,32]. However, in neurons, the formation of ɣH2AX foci is slower than in proliferating cells [33]. The signal is ultimately transduced from ATM to downstream kinases, including checkpoint kinase 2 (Chk2), thus enforcing checkpoint activation [34] to engage downstream proteins, such as p53 [35]. 

**Figure 2:**
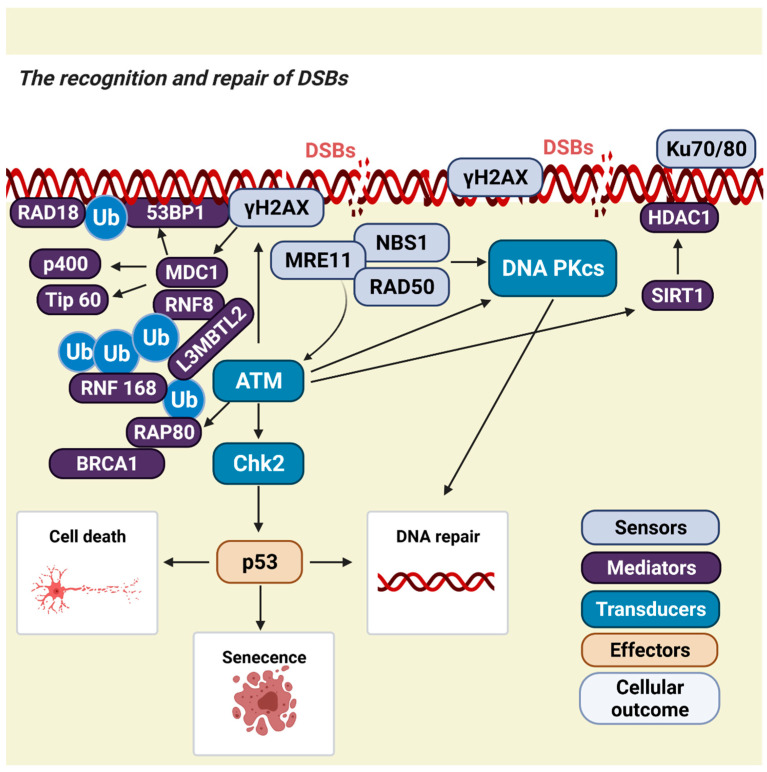
Schematic diagram illustrating the recognition and repair of DSBs, leading to either neuronal survival or death, and/or possibly senescence. Following DSB formation, the DDR is co-ordinated by multiple DNA damage sensors, transducers, mediators, and effectors. DSBs are detected by the MRN or Ku70/80 (sensor) complexes to recruit and activate transducer ATM or activate p53 (effector), facilitated by MDC1 and 53BP1 (mediators). There is also evidence that neurons persisting for prolonged periods after their initial attempts to re-enter the cell cycle undergo senescence [31,32,37].

In cycling cells, another kinase, ATR, is activated by single-stranded DNA and stalled DNA replication forks during the S phase of the cell cycle. However, the ATR-Chk1-p53 pathway is suppressed in neurons [36]. This leaves ATM and DNA-PK as the key candidate kinases that perform the same roles in neurons as in proliferating cells [36] (Figure 2).

### 2.3. DNA Repair Mechanisms in Neurons

There is a repertoire of DNA repair pathways that respond to specific types of DNA damage, SSBs, or DSBs. Importantly, the type of DNA lesion dictates the precise biochemical repair pathway activated. SSBs rely on the mismatch repair (MMR), nucleotide excision repair (NER), or base excision repair (BER) pathways. DSBs can be repaired by either homologous recombination (HR) or non-homologous end-joining (NHEJ) mechanisms in most cell types. However, neurons lack HR, and thus rely almost exclusively on the more error-prone NHEJ or SSB repair mechanisms [38]. Defects in these latter mechanisms can therefore severely impact on neuronal viability. 

The MMR pathway is involved in the recognition and correction of incorrectly paired nucleotides, resulting in excision of a large fragment of DNA from the mismatched strand and the synthesis of new DNA. Mismatches can occur in DNA due to the incorrect incorporation of nucleotides by DNA polymerases, by damage to nucleotide precursors in the cellular nucleotide pool, or by spontaneous deamination of 5-methylcytosine to thymine (G–T mismatch) or cytosine to uracil (G–U mismatch) [39]. In contrast, NER removes a large variety of DNA lesions from the genome that distort the helix and interfere with base pairing. These lesions generally obstruct transcription and normal DNA replication [40]. Finally, BER is the primary mechanism that repairs small base lesions resulting from damage induced by oxidation and alkylation [41,42].

DSBs are particularly hazardous to a cell, because they can activate apoptosis or lead to chromosomal rearrangements. HR requires a sister chromatid for error-free repair, so it normally occurs during the S or G2 phases of the cell cycle. Hence, this mechanism is not thought to be present in neurons [38]. In contrast, NHEJ involves direct ligation of the processed ends of the DNA break, so it can occur at any stage of the cell cycle [43,44]. 

Here we give only a brief overview of DNA repair mechanisms. For further molecular details of each pathway, the reader is directed to several recent and more comprehensive reviews on this topic [37,45,46]. 

## 3. Neuronal Senescence

Cellular senescence is an important mechanism implicated in many physiological and pathological processes, that is closely linked to DNA damage [47]. Senescence is also strongly implicated as a major driver of the aging process [20]. Whilst it was originally thought to be restricted to dividing cells (‘replicative senescence’), it is now well established that neurons (and other cell types) also undergo senescence in response to stress (‘stress-induced premature senescence’) [48,49,50,51]. This new field of neuronal senescence is now growing rapidly and is recognised by the International Cell Senescence Association (ICSA, [49]). 

Neurons display remarkable longevity, and in a normal adult, most remain alive despite residing in an environment of increasing stress during the aging process. The exceptional survival of neurons implies they have high resistance to apoptosis. Whilst this is poorly understood, induction of senescence in neurons, triggered by cell stress during aging, has been implicated in longevity [4,24]. In addition, as terminally differentiated cells, neurons inherently display one feature of senescence: cell-cycle arrest. Unrepaired DNA damage is linked to senescence and apoptosis in proliferating cells [52]. However, the relationship between DNA damage and neuronal senescence is poorly studied, and similarly, the contribution of senescence to neuronal death remains largely unexplored. 

## 4. Re-Entry of Neurons into the Cell Cycle

In proliferating cells, induction of the DDR temporarily arrests the cell cycle. In this process, activation of the DNA damage checkpoint inhibits the entry of cells containing damaged DNA into mitosis, thus blocking the cell cycle at G2/M. This provides time for the DDR to repair the lesion [53]. Hence, DNA damage and the cell cycle are closely linked in proliferating cells. In contrast, fully mature adult neurons were previously thought to remain in the G0 phase of the cell cycle indefinitely. However, increasing evidence suggests that neurons do re-enter the cell cycle in response to DNA damage, although this does not represent the full cycle, since re-entry is most frequently observed in the G1 to S phases [54].

Initial evidence for a link between the DDR and cell cycle re-entry in neurons was obtained from studies involving low levels of DNA damage induced by ROS. This required phosphorylation of pRb by cyclin C to allow cells to initiate G1 and activate NHEJ. Furthermore, blocking the initiation of G1 prevented activation of NHEJ, and thus DNA repair [55]. A similar scenario was observed in another study involving induction of DNA damage in rat neurons by ionizing radiation. More DSBs and increased expression of cyclin D were accompanied by upregulation of the cell cycle inhibitor p21, which arrested the cycle in G1 and prevented entry into the S phase, allowing repair of DNA by NHEJ [56]. 

Normally, once DNA damage is sensed and repaired, neurons return to the G0 phase of the cell cycle. However, with more severe DNA damage a different scenario was observed, whereby neurons entered the S phase of the cycle, with induction of DNA replication, cyclinE-Cdk2 expression, and eventually apoptosis [57]. In contrast, entry of neurons into the M phase of the cell cycle has not yet been observed. This is not surprising because cell-cycle re-entry largely results in apoptosis before cells can enter the M phase, thus it is likely to lead to mitotic catastrophe [58].

Whilst it is conventionally regarded that re-entry of neurons into the cell cycle renders them more susceptible to apoptosis and hence neurodegeneration, a recent study challenged this viewpoint. Mature hippocampal neurons re-entering the cell cycle were found to be protected from exposure to amyloid β (Aβ) and cell death, in contrast to those that did not re-enter the cell cycle [59]. However, this study did not examine how DNA damage was related to the cell cycle re-entry of neurons. Further studies are therefore required to elucidate the complex relationship between DNA damage, the cell cycle, and apoptosis in neurons.

## 5. Mitochondria and DNA Damage in Neurons

Mitochondria are important for many neuronal functions, including ATP production, generation of reactive oxygen species (ROS), Ca2+ signalling, membrane excitability, neurotransmission, and plasticity [60]. During aging, mitochondrial function and DNA repair declines, and ROS accumulate [61]. Mitochondrial DNA repair pathways are less efficient than those in the nucleus, and mitochondrial DNA damage is implicated in aging and neurodegeneration [62].

## 6. Cell Death Mechanisms Relevant to DNA Damage

Cell death is an important physiological mechanism that maintains homeostasis of the organism, and it is also significant in pathological situations. Interestingly, the cell death field has expanded greatly in recent years, and many novel pathways have been identified, several of which are associated with DNA damage. The Nomenclature Committee on Cell Death (NCDD) recently (2018) formulated guidelines for the definition and elucidation of cell death mechanisms, which will be referred to in this review [63].

The NCCD defines dead cells as those that either display irreversible plasma membrane permeabilisation or have undergone complete fragmentation [64]. The recommendations describe two broad, mutually exclusive categories: ‘accidental’ and ‘regulated’ cell death. Accidental cell death (ACD) refers to the structural disassembly of cells following exposure to harsh physicochemical conditions and does not involve specific molecular machinery. Whilst it can occur in vivo following burns or traumatic injuries, this form of cell death cannot be prevented or modulated. It is therefore not considered to be a target for therapeutic intervention. In contrast, regulated cell death (RCD) involves genetically encoded molecular machinery that can be targeted by pharmacologic and/or genetic interventions [65]. 

RCD can be further categorised into a growing number of types. Historically, this was based on morphological features associated with each process. However, the updated guidelines now classify these types according to their molecular characteristics, although it is important to recognise that the RCD pathways are highly inter-related. Nevertheless, not all mechanisms have been described in neurons or in relation to DNA damage. Table 1 summarises the major RCD classifications recognised by the NCDD. It also includes novel death mechanisms only recently described (oxeiptosis, alkaliptosis), and thus absent from the NCDD classifications, and atypical necrosis induced by transcriptional repression (TRIAD) cell death, which has been recently associated with Huntington’s disease pathology. In this review, however, we will discuss only those RCD types relevant to DNA damage in neurons. Nevertheless, the reader is referred to a recent review that describes the other cell death mechanisms in detail [66].

### 6.1. Cell Death in Neurons

Despite accumulating damage to proteins, lipids, DNA, and organelles during their lifetime, neurons need to survive and maintain their appropriate circuits for decades. RCD occurs physiologically during embryonic and early postnatal development in neurons, and pathologically in neurodegenerative disorders [67]. Several of the cell death mechanisms detailed above have been described in neurons, but there is also extensive crosstalk between these processes [67]. Furthermore, neuronal cell death is often aided through interactions with neighbouring neuronal and glial cells by ‘non-cell autonomous’ mechanisms [67]. Neurons of the same type are also highly heterogenous and can display variations in the morphology and architecture of their dendritic arbors, axons, or synaptic connections, with differences in expression levels and localisation of neurotransmitters and their receptors. It is likely that each neuron in a degenerating population responds to its own unique microenvironment, and that this heterogeneity shapes the probability of those neurons to die death [68]. Below we discuss the different types of RCD relevant to neurons in response to DNA damage.

### 6.2. Apoptosis

Apoptosis is essential for development and homeostasis, and it has been widely studied as an RCD response to DNA damage. It needs to be tightly regulated because once apoptosis commences, death of the cell is almost inevitable [69,70,71,72]. The two best understood apoptotic mechanisms are the intrinsic and extrinsic pathways. The intrinsic pathway is activated from within the same cell by intracellular signals from mitochondria generated in response to cell stress. In contrast, the extrinsic pathway is activated by extracellular signals from other cells that bind to cell surface receptors. Both pathways result in activation of initiator caspases that subsequently initiate executioner caspases, which then kill the cell [73].

The intrinsic apoptotic pathway is activated following irreparable DNA damage, depending on the cellular context. It results in mitochondrial outer membrane permeabilisation (MOMP), in which the outer mitochondrial membrane forms apoptotic pores that allow proteins to pass. MOMP is tightly regulated by interactions between the Bcl-2 family of proteins, both pro-apoptotic (such as Bcl-2-associated X protein (Bax) and Bcl-2 homologous antagonist/killer (Bak)) and anti-apoptotic members (including B-cell lymphoma 2 (Bcl-2) and B-cell lymphoma extra-large (Bcl-XL)). Bax/Bak oligomerisation is an important step in MOMP that results in the release of mitochondrial cytochrome c to the cytoplasm. This triggers the formation of the apoptosome, a large, multimolecular complex consisting of pro-caspase-9, apoptotic protease activating factor 1 (Apaf-1), and cytochrome c. This complex results in activation of the initiator caspase, caspase 9, and then of downstream executioner caspases (-3, -6 and -7) that induce cell death. 

After induction of apoptosis, a select handful of nucleases have been implicated in apoptotic DNA fragmentation, and caspase-activated DNase (CAD) is the most characterised of these [74]. In differentiated myoblasts, caspase 3/CAD-mediated DNA strand breaks are a prerequisite for inducing cell differentiation [75]. Caspase activation of CAD participates in the formation of transient genome-wide DNA damage/strand breaks during the early stages of skeletal muscle differentiation. Reduction in the expression of CAD leads to the absence of DNA damage with a near complete blockade of differentiation [75]. However, this process has not been described in neurons. Caspase-7 inhibits DNA repair by digesting PARP, and caspase-3 degrades DNA-PK, resulting in neuronal death [76]. 

Many forms of DNA damage activate p53, a multi-functional protein that integrates various stress signals into diverse cellular responses, including activation of apoptosis. Importantly, it acts as a ‘guardian of the genome’ by regulating a variety of DNA repair and DDR mechanisms. In response to DNA damage, p53 regulates cytochrome c release by inducing permeablisation of the outer mitochondria membrane or by transcriptional induction of pro-apoptotic Bcl-2 proteins, including Bax, p53 upregulated modulator of apoptosis (PUMA), and phorbol-12-myristate-13-acetate-induced protein 1, also known as NOXA [77,78,79]. In fibroblasts and several cell lines, following DNA damage, the release of cytosolic cytochrome c induces a rapid, caspase-dependent apoptotic death. This result implies that the cytosolic accumulation of cytochrome c alone is sufficient to activate caspases in these non-neuronal cells [80]. In contrast, in post-mitotic cells, including neurons, although cytochrome c is necessary, it is not sufficient to induce death due to the presence of X-linked inhibitor of apoptosis (XIAP), a potent inhibitor of caspases. In this case, an additional step mediated by p53 is required: induction of Apaf-1. This in turn overcomes XIAP inhibition, and thus allow cytochrome c to induce apoptosis [81,82].

### 6.3. Autophagy-Dependent Cell Death

Autophagy-dependent cell death can be also triggered by damage to DNA. Macroautophagy (referred to simply as ‘autophagy’ hereafter) is defined as the degradation of intracellular components, including organelles and long-lived proteins, by engulfment in double-membrane vesicles, or ‘autophagosomes’ [83,84]. Autophagosomes then fuse with lysosomes, in which their contents are degraded and can then be recycled [83]. Depending on the cellular context, type, and magnitude of stress, autophagy can be a pro-survival adaptive mechanism, or it can induce autophagy-dependent cell death [85,86]. Furthermore, depending on the stimulus and cellular context, in some circumstances, autophagy may antagonize or delay apoptosis, or if these two processes act exclusively, they serve as reciprocal backup mechanisms [87]. The specific ablation of autophagy in neurons leads to neurodegeneration, demonstrating the existence of a specific relationship between autophagy and neuronal viability [88]. 

Previously, autophagy and DNA damage were regarded as being independent processes. However, evidence has increasingly indicated that autophagy and DNA repair are closely related events [89], despite these processes occurring in different cellular locations (the cytoplasm and nucleus, respectively). Extensive interactions have been identified between these two mechanisms, and this interchange is thought to occur in both directions. Many studies have shown that autophagy is stimulated as a protective mechanism following DNA damage [80,90,91,92,93,94]. Furthermore, multiple DNA repair pathways, including HR, BER, NER, and MMR, are regulated by autophagy [95]. Inhibition of p53 leads to enhanced autophagy in cell lines (including SH-SY5Y neuroblastoma cells), thus improving the survival of cells defective in p53 by allowing them to maintain high levels of ATP. This finding might be of relevance in neurons where the expression of p53 is strongly decreased and basal autophagy is activated [96,97]. However, it remains unclear whether this process is modulated by DNA damage.

### 6.4. Necrosis: Necroptosis and MPTP-Dependent Regulated Necrosis

Necrosis is a regulated form of cell death usually initiated following energy loss. At least two types of programmed necrosis in response to DNA damage have been described: necroptosis and mitochondrial permeability transition pore (MPTP)-dependent regulated necrosis. 

#### 6.4.1. Necroptosis

This type of RCD displays features of both apoptosis and necrosis and was therefore named ‘necroptosis’. It is associated with inflammation, and can be initiated by several stimuli, including DNA damage, although activation by tumour necrosis factor receptor 1 (TNFR1) is the most characterised mechanism. Necroptosis involves sequential activation of receptor-interacting protein kinase 1 (RIPK1), RIPK3, and mixed-lineage kinase domain-like protein (MLKL) [63]. RIPK 1 interacts with RIPK 3, Fas-associated protein with death domain (FADD), caspase-8, and cellular FLICE-inhibitory protein (cFLIP) isoform [98] to form a complex named the ‘necrosome’ or ‘ripoptosome’. Phosphorylation of RIPK1 and then RIPK3 allows the recruitment of MLKL to the necrosome. Phosphorylation of MLKL by the necrosome leads to its oligomerisation and insertion into membranes of organelles and the plasma membrane [99,100,101,102]. This induces lysis by incompletely understood mechanisms [99]. Importantly, induction of necroptosis is possible only when caspase-8 is inhibited by either pharmacological caspase inhibitors or virally expressed proteins [103]. This is the case in primary microglia cells [104,105]. However, conflicting findings have been obtained in neurons. In a HT22 hippocampal cell line and primary cortical neurons, caspase 8 inhibition was required for induction of necroptosis. In contrast, in primary cerebellar granule neurons and forebrain neurons, caspase inhibition did not lead to necroptosis after stimulation of TNF receptors [98,104,106,107]. Since RIPK1 plays a crucial role in determining cellular fate by triggering necroptosis after extensive DNA damage in cancer cell lines [108] and in certain post-mitotic neuronal populations [107], it would be interesting to examine whether DNA damage modulates necroptosis in neurons. However, this possibility has not been demonstrated experimentally yet.

#### 6.4.2. MPTP-Dependent Regulated Necrosis

MPTP-dependent regulated necrosis is a form of RCD initiated by specific perturbation of the intracellular microenvironment, such as severe oxidative stress and cytosolic Ca2+ overload. To date, peptidylprolyl isomerase F (PPIF; best known as cyclophilin D, CYPD), is the only protein that has been validated as a requirement to induce MPT in vivo. In neurons, increased production of ROS was followed by increased Ca2+ concentrations and elevated CYPD, which was associated with mitochondrial and neuronal degeneration [109,110]

### 6.5. Parthanatos

PARP-1-dependent cell death, or ‘parthanatos’, is a unique cell death pathway distinct from apoptosis, necroptosis, and autophagy -dependent cell death. It involves excessive activation of PARP-1, leading to elevation of long-chained and branched PAR polymers that covalently attach to acceptor proteins, including PARP1 itself, histones, various DNA repair proteins, and transcription-related factors [111,112,113]. Therefore, parthanatos refers to cell death resulting from the toxic presence of PAR [114,115]. It can be triggered by oxidative stress, nitric oxide (NO) production, or extensive DNA damage. DNA damage induced by several mechanisms is known to result in overactivation of PARP-1, producing PAR polymers and thus parthanatos. This includes ionizing radiation, exposure to N-methyl-D-aspartate (NMDA) or DNA-alkylating agent N-methyl-N0-nitro-N-nitrosoguanidine (MNNG), oxygen glucose deprivation, or prolonged treatment with hydrogen peroxide or other forms of ROS. 

Parthanatos exhibits features commonly associated with both apoptosis and necrosis. However, parthanatos was not specifically defined until it was discovered that apoptosis-inducing factor (AIF) is a mediator of caspase-independent cell death that acts downstream of PARP-1 following DNA damage [112,114]. Moreover, increased PARP1 activity not only facilitates the release of AIF, but also the loss of mitochondrial transmembrane potential, ultimately leading to necrotic cell death [116]. 

After DNA damage, PAR polymers accumulate at sites of damaged DNA, resulting in the release of PAR from the nucleus into the cytoplasm. It then stimulates the release of mitochondrial AIF [117] which translocates to the nucleus, leading to large-scale DNA fragmentation. The translocation of mitochondrial AIF can be prevented by the PAR-degrading enzyme PAR glycohydrolase (PARG), or by ADP-ribosyl-acceptor hydrolase 3 (ARH3), but not by caspase inhibition [118,119]. Since AIF itself does not possess nuclease activity, AIF binds and transports macrophage migration inhibitory factor (MIF) to the nucleus. MIF is broadly expressed in multiple cell types, including neurons in the brain [120,121], and it possesses DNA nuclease activity that cleaves genomic DNA into large (approximately 50 kb) fragments, leading to chromatin fragmentation. 

### 6.6. Ferroptosis

Similar to other forms of cell death, ferroptosis displays distinct biochemical, morphological, and cellular features. It is initiated by perturbation of glutathione-dependent antioxidant defence systems, resulting in extensive lipid peroxidation and eventual cell death. It is characterised by iron dependency, and it is induced by inactivation of glutathione peroxidase 4 (*GPX4*) [122]. Moreover, the DDR is critical to ferroptosis. In many cell types, including neurons, GPX4 prevents excessive lipid peroxidation in a glutathione-dependent manner. Similarly, inhibitors of GPX4 [123] and knockout of *GPX4* in vivo induces ferroptosis. ROS and protein aggregation can trigger neuroinflammation, which induces the release of pro-inflammatory cytokines that stimulate iron uptake by neurons, inducing ferroptosis [124]. Interestingly, conditional deletion of *GPX4* from forebrain neurons in mice results in neurodegeneration and cognitive dysfunction. This implies that ferroptosis is associated with neurodegenerative conditions such as Alzheimer’s disease [125]. Ferroptosis has also been detected in induced pluripotent stem cell (iPSC)-derived motor neurons, suggesting that it might play a biologically important role in these cells [126]. However, ferroptosis has not yet been described in relation to DNA damage in neurodegenerative disorders.

Recently, both ATM and ATR emerged as the top hits from genetic screening experiments performed to identify kinases essential for cell death triggered by cysteine deprivation, which induces ferroptosis [127]. Moreover, several ATM inhibitors also rescued cancer cells from ferroptosis, although this was unrelated to canonical ATM targets Chk2 and p53. It is also important to note that these experiments were performed in the absence of DNA damage. The interplay between ferroptosis and DNA damage in neurons was recently described in an in vitro human model of neuroferritinopathy (NF) [128]. The iPSC-derived neurons exhibited increased cytosolic iron, detectable as ferritin aggregates, oxidative damage, and the onset of a senescence phenotype. NF cells also displayed impaired survival and underwent death by ferroptosis. Moreover, increased Ser139 phosphorylation on H2AX in NF neurons compared to control cells was observed, indicating the presence of DNA damage. 

### 6.7. Pyroptosis

Pyroptosis is a form of RCD driven by activation of the inflammasome. Part of the innate immune system, the inflammasome is a cytosolic multi-protein complex that induces inflammation and regulates the activation of pro-inflammatory caspases. It is responsible for the release of interleukin (IL)-1 family members, the formation of apoptosis-associated speck like protein containing a CARD domain (ASC) specks, and activation of pro-inflammatory caspases [129]. Whilst pyroptosis shares some features associated with apoptosis, pyroptosis involves caspases that possess both initiator and effector functions. It is therefore biochemically different from apoptosis [130]. 

Two pathways are known to trigger pyroptosis. First, the canonical inflammasome pathway requires cleavage of procaspase-1 to activate caspase-1 within the inflammasome. Once activated, caspase-1 can proteolytically cleave pro-interleukin (IL)-1β to IL-1β and cleave pro-IL-18 to IL-18 to induce inflammation. The second, or non-canonical inflammasome pathway, employs caspase-4, -5, and -11 as cytosolic sensors of cytosolic lipopolysaccharide (LPS) [131]. Gasdermin D (GSDMD) is a crucial component of inflammasomes in both the canonical and non-canonical pathways. It is required for the secretion of IL-1β and it is a substrate of inflammatory caspases (caspase-1/4/5/11). Furthermore, the cleaved N-terminal domain of GSDMD oligomerizes to form cytotoxic pores on the plasma membrane [66].

Recently, it was demonstrated in a mice model deficient in GSDMD that DNA damage surveillance by the inflammasome is required for normal brain development and function [132]. Disruption to this pathway led to the development of anxiety-related behaviours, DNA damage accumulation in the CNS, and increased numbers of neurons in the adult brain. However, the functional consequences of the observed increase in DNA damage requires further elucidation.

### 6.8. Lysosome Dependent Cell Death

Lysosomal cell death (LCD) is a type of RCD executed by hydrolytic enzymes released into the cytosol following lysosomal membrane permeabilisation (LMP). Lysosomes are acidic cellular organelles that can degrade a variety of heterophagic and autophagic cargos. Among lysosomal hydrolases, several different cathepsins are responsible for the initiation and execution of LCD [133]. LMP occurs when cells are exposed to lysosomotropic detergents and ROS. Likewise, several signals have been shown to induce LMP, including lysosomal p53 [134,135], phospholipase A2 activity [136], lysosomal pore formation by Bax [137], cleavage of the lysosomal membrane protein Lamp2 [138], and DNA-damage-regulated autophagy modulator 1 (DRAM1) [139]. LMP can be triggered by events that also activate other cell death pathways and cross-react with other cell death mechanisms. Therefore, a systematic investigation of the causes of LMP in neurons and how LCD interplays with DNA damage in these cells is required [140]

## 7. Mechanisms of Cell Death Induced by DNA Damage in Neurodegenerative Diseases

Neurodegenerative diseases are a heterogeneous group of conditions characterised by the progressive degeneration of specific groups of neurons. The most common pathological hallmark is the aggregation of misfolded proteins in affected tissues, forming either intracellular or extracellular inclusions [141]. Whilst the types of neurons affected and the specific protein deposited varies in each disease, inclusion formation is a prominent and common feature. Similarly, these disorders also show overlap in the disease mechanisms implicated in pathogenesis. Most of these conditions arise sporadically, with no previous family history, although a smaller proportion of cases are familial and associated with specific mutations.

Abnormalities in RCD are widely described and associated with pathophysiology and loss of specific types of neurons in neurodegenerative diseases. Several of these RCD mechanisms are known to be induced by the DDR. Moreover, the accumulation of DNA damage and deficient DNA repair are increasingly implicated in the pathogenesis of these conditions. Interestingly, dysfunctional DNA repair is also linked mechanistically to other disease processes implicated in neurodegeneration, including mitochondrial dysfunction, redox dysregulation, and defects in protein quality control [142,143,144]. In addition, aging is the biggest risk factor for neurodegenerative disorders [145,146], and incomplete maintenance of genome integrity is widely implicated in the accumulation of DNA damage during aging [147]. Furthermore, since DNA is the utmost hierarchical level of biological information, unrepaired lesions in post-mitotic neurons will lead to significant abnormalities and mutations. However, to date, most studies examining the role of DNA damage in neurodegeneration have not described the specific mechanisms by which neurons die in each condition. Similarly, an increasing number of RCD mechanisms are implicated in neuronal death in neurodegenerative diseases, but few studies have linked these events to DNA damage. 

Below we discuss our current understanding of how RCD mechanisms are associated with DNA damage in neurodegenerative diseases. We focus specifically on those studies in which precise neuronal death mechanisms were described following DNA damage in each condition. Some of these studies showed a direct relationship between cell death and the DDR, but for others, the link was more tenuous, including apoptosis, parthanatos, and necrosis. We do not provide a detailed analysis of the role of DNA damage in neurodegeneration here. For a comprehensive review of this topic, independent of cell death, please see several excellent recent reviews [7,144,148,149].

### 7.1. Alzheimer’s Disease (AD)

Alzheimer’s disease (AD) is the most common neurodegenerative disorder. It is characterised by the progressive loss of memory and neuropsychiatric symptoms, resulting from loss of cortical neurons (located in the locus coeruleus and nucleus basalis of Meynert) in the brain [150,151]. Several mechanisms are implicated in the pathophysiology of Alzheimer’s disease, including Aβ deposition [152], tau hyperphosphorylation [152], neuroinflammation [150], constriction of brain capillaries [153], Aβ interaction with hippocampal ghrelin/GHSR1α signalling [154], DNA damage [155], disrupted RNA hemostasias [156], mitochondrial dysfunction [157], endoplasmic reticulum (ER) stress [158], and proteostasis dysfunction [159]. The pathological hallmarks of AD [160] are deposition of extracellular Aβ plaques and intracellular neurofibrillary tangles (NFTs). Aβ is produced by cleavage of the amyloid precursor protein (APP) by the gamma secretase complex, which includes presenillin-1 [161]. Growing evidence suggests that Aβ peptides accumulate intraneuronally before extracellular plaque deposition [162]. Several mutations are present in AD cases, including in the genes encoding APP (*APP*) and presenilin 1 and 2 (*PSEN1* and *PSEN2*). Furthermore, the genotype of the apolipoprotein E (*APOE*) gene is a major risk factor for AD [163].

There is extensive evidence for DNA damage in AD [155,164,165]. In post-mortem AD patient brains, more DSBs and reduced expression of DSB repair proteins compared to tissues from control patients has been reported [166]. Decreased DSB repair activity, reflecting impaired NHEJ, was also detected in the AD brains [167]. Studies in transgenic mouse models (calcium/calmodulin-dependent protein kinase II (CK)CK-p25 and tau P301S mice) detected elevated DNA damage during the pre-symptomatic disease stages, prior to the presence of neurological symptoms or neurodegeneration [168,169]. This evidence reveals that DNA damage is implicated early in pathophysiology in mouse models of AD, implying that it has an active role in neurodegeneration. In addition, DNA damage has been detected at symptom onset in another mouse model of AD (3xTgAD) [170], implying it may also contribute to disease progression. Deficiency in the BER enzyme DNA polymerase β was associated with apoptosis in the same model (3xTgAD), which displayed both Aβ and tau pathology [170]. Aβ_25–35_ also inhibited DNA DSB repair pathways in PC12 cells [171]. Furthermore, oxidative DNA damage was detected in the cerebrospinal fluid and peripheral cells of patients with AD or mild cognitive impairment in the brain. [172,173,174]. In another study, DSBs were detected in susceptible neuronal and glial cell populations in human post-mortem AD tissues. [164]. DNA damage was also associated with somatic recombination of the *APP* gene [175]. 

In AD, several RCD mechanisms are implicated in neuronal death, including apoptosis, parthanatos, ferroptosis, necrotic cell death, and autophagy. However, only apoptosis, parthanatos, and necrotic cell death have been described in relation to DNA damage. We discuss this evidence below.

#### 7.1.1. DNA Damage and Apoptosis in AD

DNA damage and apoptosis were linked in an early study of AD patient brains, in which terminal deoxynucleotidyl transferase (TdT) labelling for DNA strand breaks in neurons was detected by immunoreactivity for c-Jun [176]. Induction of c-Jun has also been reported in neurons exposed to Aβ, consistent with apoptosis [177,178]. More recently, enhanced production of Aβ1-42 in neurons was linked to induction of mild DNA damage [179].

The levels of 8-oxoguanine (8-oxoG) DNA glycosylase are reduced in the hippocampus of AD brains compared to controls, implying that BER is defective in these tissues [180]. Similarly, BER defects, including decreased activity of DNA glycosylase and reduced DNA synthesis by DNA polymerase β, were reported in AD brain tissues [181]. Increased apoptosis has been reported in mice lacking DNA polymerase β [182]. Furthermore, the activity of components of BER were inhibited in mitochondrial lysates of AD brains [183]. In addition, mutations in the gene encoding 8-oxoG DNA glycosylase (*OGG1*) were detected in AD patients, resulting in reduced enzymatic activity [184]. Taken together, these findings reveal decreased activity of different DNA glycosylases in AD, leading to defective DNA BER and neurodegeneration. If DNA damage is prolonged, persistent ATM/p53 elevation upregulates apoptotic proteins, triggering cell death [52]. 

Neural stem cells (NSCs) are multipotent cells that mediate neurogenesis in restricted regions of the adult brain. Efficient DNA repair is an important process in these cells, allowing the genome to remain intact in new neurons. However, impaired neurogenesis has been linked to neuronal death in neurodegenerative disorders, including AD [185]. Both NSC proliferation and differentiation can be inhibited by oxidative DNA damage and associated BER DNA repair mechanisms [186]. Furthermore, BER and DSB repair by SIRT6 is protective in AD due to accelerating neurogenesis of hippocampal neurons [187]. Increased apoptosis and tau phosphorylation have been reported in SIRT6 knockout mice following the induction of DNA damage [188]. Suppression of the INK4A/ARF locus, which encodes two essential tumour suppressors (p16INK4A and p14ARF), facilitates this process [189,190]. 

B lymphoma Mo-MLV insertion region 1 homologue (*BMI1*) is an oncogene encoding a protein linked to both DNA repair and apoptosis, RING finger protein 51 (RNF51). It can promote DSB repair via both HR and NHEJ [191,192], and it regulates apoptosis and senescence by suppression of the p19Arf/p53 and p16Ink4a/pRb tumour-suppressor axes [193]. Expression of *BMI1* is decreased in post-mortem AD brains compared to control tissues [193]. Heterozygous mice lacking one allele of *BMI1* develop cognitive impairment, tau phosphorylation, Aβ plaque accumulation, decreased neuronal density, and significant apoptosis in the hippocampus [193]. Furthermore, Aβ plaques accumulate and tau hyperphosphorylation is present in *BMI1* knockout human neurons [194]. Together, this evidence links apoptosis, DNA repair, and neurodegeneration in AD. 

Breast cancer type 1 (BRCA1) is a multi-functional and central protein in the DDR [195,196]. In iPSCs derived from familial AD patients, BRCA1 expression was increased, and abnormalities in presenilin1 were detected. This was associated with increased CDC 25C phosphorylation and elevated levels of Aβ pathology, implying that neurons with increased BRCA1 re-enter the cell cycle and undergo apoptosis [197]. 

DNA fragmentation has been detected using TUNEL assays using brain tissues obtained from AD patients, further linking DNA damage to apoptosis [198]. TUNEL-positive cells can appear with an apoptotic-like morphology, including granulated and marginated chromatin, a shrunken, irregular cellular morphology and apoptotic bodies. However, TUNEL reactivity cannot be taken as a specific indicator of apoptosis alone, unless specific morphological characteristics of apoptosis are also present. Here, the TUNEL-positive cells displayed few or none of the classical morphological characteristics of apoptosis [199], indicating that necrosis in AD is also involved.

#### 7.1.2. DNA Damage and Parthanatos in AD

As well as apoptosis, there is also significant evidence that DNA damage induces parthanatos in AD. Oxidative stress and PARP1 upregulation are present following accumulation of Aβ peptides in the hippocampus of adult rats [200]. Increased levels of both PARP1 and PAR were detected in neurons of human post-mortem AD brains [201,202]. Similarly, PARP1 upregulation has been reported in brains of AD patients, predominantly in the frontal and temporal lobes [203]. Furthermore, reduced levels of nuclear PARP1 are present in the hippocampus of human AD brains [204]. Nicotinamide adenine dinucleotide (NAD) has emerged as a major moderator that controls energy metabolism, mitochondrial function, aging, and cell death [205]. In addition, administration of exogenous NAD reduced the toxic effects of Aβ and increased the levels of PARP1 in primary rat cortical neurons [205]. NAD+ treatment was also protective against Aβ-induced DNA damage in primary rat cortical neurons [205]. 

PARP1/PAR colocalised with Aβ, tau, and microtubule-associated protein 2 in AD brain tissues [206]. Furthermore, PARP1 upregulation also enhanced deposition of Aβ and the formation of tau tangles in SH-SY5Y cells [207]. Since PARP1 activation has been detected both upstream and downstream of Aβ, activation of PARP1 is implicated as an early and essential event in the pathogenesis of AD [208]. Furthermore, PARP1 reduction in the nucleus, leading to activation of DNA cytosine-5-methyltransferase 1 and silencing of nucleolar rRNA gene (rDNA), was implicated as an early marker of cognitive impairment in AD [209]. These studies therefore provide evidence that dysregulation of PARP1 is a consistent feature of AD, thus implicating parthanatos in neuronal death in this condition.

#### 7.1.3. DNA Damage, p53 and Neuronal Death in AD

As detailed above, p53 is implicated widely in multiple cell death pathways related to DNA damage. Furthermore, several studies have shown that p53 is involved in the death of neurons in AD [210]. However, whilst this implies that DNA damage is linked to the pathophysiology of AD, most of these studies did not directly examine the relationship between the DDR and p53-associated RCD. 

p53 is significantly increased in the temporal cortex of AD brains [211,212], and p53 mediates apoptosis and the release of soluble neurotoxins following exposure to Aβ peptides [213]. Whilst these studies did not specifically examine DNA damage in relation to p53, one recent report did provide evidence of this relationship in AD. p53 is known to aggregate and cross-seed in vitro, and these aggregates were detected in human AD brains and mouse models based on tau [214]. Oligomers and fibrils of p53 also interacted with tau aggregates, and a p53-mediated DDR was found to be impaired in AD, implying that loss of p53 function in the nucleus is involved in AD pathogenesis [214]. Intracellular Aβ1–42 is also selectively cytotoxic to human neurons through the p53–Bax cell death pathway [215]. Interestingly, p53-mediated apoptosis has also described in glial cells in AD. Whilst microglia can be either protective or harmful in AD, altered microglial responses to Aβ are linked to an increased risk of AD [216]. Hence, although these studies do not directly examine DNA damage and p53-mediated RCD in AD, they imply that p53 is centrally involved in neuronal death in this disorder.

### 7.2. Parkinson’s Disease

The second most common neurodegenerative disease after Alzheimer’s disease is Parkinson’s disease (PD) [217,218]. The main feature of this condition is the pronounced loss of dopamine-producing neurons in the substantia nigra pars compacta (SNpc) [217]. This leads to a significant depletion of dopamine (DA) in the striatum [219], where these neurons project. Hence, PD is characterised by severe motor symptoms, including resting tremors, postural imbalance, bradykinesia (slowness of movement), and rigidity [220,221,222]. Several mechanisms are implicated in the pathophysiology of PD, including accumulation of α-synuclein in various parts of the brain [223] (primarily in theSNpc) and degeneration and subsequent loss of dopamine [223], mitochondrial dysfunction [224], oxidative stress, and neuroinflammation [225,226]. The major pathological hallmark of PD is the presence of abnormal cytoplasmic Lewy bodies containing α-synuclein [227]. More than 23 mutations have been linked to PD, including in the genes encoding α-synuclein, parkin, DJ-1, and PINK-1 [228].

There is extensive evidence for DNA damage in PD [229,230]. Age-related increases in DNA damage, as well as energy deficits and oxidative stress, have been observed in dopaminergic neurons following exposure to specific environmental and genetic factors [231]. DNA damage has been detected at symptom onset in animal models of PD [232], implying it may contribute to disease progression. DNA damage and associated death of dopamine neurons are also present in brain tissues from PD patients [233]. A growing body of evidence also suggests that both oxidative and nitrative stress induce DNA damage in PD [234,235]. 

Several RCD mechanisms have been linked to neuronal death in PD, including extrinsic and intrinsic apoptosis, necrosis [236], ferroptosis [237] parthanatos, anoikis [238], autophagic cell death [239], and pyroptosis [240]. However, only apoptosis and parthanatos have been described in relation to DNA damage. We discuss this evidence below.

#### 7.2.1. DNA Damage and Apoptosis in PD

It is well established that neurons can die by apoptosis in PD, although this has not been linked specifically to DNA damage in patient tissues. However, DNA fragmentation following induction of caspase-3 activity has been demonstrated in both in vitro and in vivo PD models [241]. In PD patient tissues, a positive correlation between the proportion of caspase-3-positive neurons and the extent of dopaminergic neuron loss was detected, implying that SNpc neurons expressing caspase-3 are more prone to undergo apoptosis [242]. Likewise, caspase-9 and caspase-8 expression was increased substantially in nigral cells of PD patient brains compared to controls [243]. Furthermore, more neurons in the SNpc of PD patients displayed activated caspase-3 compared with controls [242,244]. Likewise, elevated expression of Bax [244] and MOMP regulator glyceraldehyde-3-phosphate dehydrogenase (GAPDH) was detected in nigral cells from PD patients [244,245]. 

A more direct link between apoptosis and DNA damage has been described in relation to α-synuclein. Overexpression of mutants α-synuclein^Ala30Pro^ or α-synuclein^Ala53Thr^ induces apoptosis in human neuroblastoma SH-SY5Y cells [246]. When localised within the nucleus, α-synuclein colocalised with γH2AX, and with PAR in human HAP1 cells and α-synuclein transgenic mouse brains [247]. Depletion of α-synuclein resulted in enhanced DSB levels and impaired DNA repair following bleomycin treatment [247]. Likewise, more neuronal DSBs were observed in α-synuclein knockout mice, indicating that α-synuclein modulates DNA repair [247]. A recent study detected more DNA damage in two PD mouse models based on α-synuclein [147] that was associated with more dopaminergic cell death by an undefined mechanism in the SNpc [147]. 

Dysfunction of mitochondria is implicated as an important pathogenic mechanism in PD, and damage to the mitochondrial genome has also been associated with this disorder [248]. Mitochondrial DNA encodes 13 essential proteins of the mitochondrial respiratory chain, and compared to nuclear DNA, the absence of histones renders it more susceptible to DNA damage induced by oxidative stress [249]. Quality-control mechanisms to remove damaged mitochondrial DNA, primarily mitochondrial fusion, fission, and mitophagy, can be induced by excessive DNA damage [250]. Increased mitochondrial fission and damage results from loss of PINK-1 function [251], and both PINK1 and Parkin are implicated in maintaining mitochondrial homeostasis. 

Somatic deletion mutations in mitochondrial DNA were detected and associated with respiratory chain deficiencies in SNpc neurons from both aged controls and PD patients [248]. Mitochondrial dysfunction, complex I inhibition, and pathology in PD has also been linked to impaired NER DNA repair [142]. Cybrids made from mitochondrial DNA from the SNpc of sporadic PD patients display reduced complex I activity [252]. PD patients also have a higher overall rate of mitochondrial DNA mutations compared to controls, although no inherited mutations have been associated with this disorder [248,253].

#### 7.2.2. DNA Damage and Parthanatos in PD

Similar to AD, there is evidence that parthanatos is induced in PD. 1-Methyl-4-phenyl-1,2,3,6-tetrahydropyridine (MPTP) is a prodrug to a neurotoxin that induces neurodegeneration of dopaminergic neurons in the SNpc (MPP+), resulting in symptoms of PD. Several studies have shown that parthanatos is involved in the neurotoxicity of MPTP, resulting in the selective death of dopamine neurons. Furthermore, it is known that DNA is fragmented following MPTP treatment both in vivo and in vitro [254,255]. Overactivation of PARP was demonstrated following MPTP administration in mice, resulting in toxicity to dopaminergic neurons [256]. Similarly, inhibition of PARP rescued MPTP-induced depletion of striatal ATP and NAD+ levels [257], and significantly attenuated MPTP-induced striatal dopamine depletion in both cultured rat primary dopaminergic neurons [258] and mice [259]. As well as MPTP, pharmacological inhibition of PARP-1 was protective against α-synuclein-induced toxicity in primary ventral neuronal cultures [258]. This result also suggests that PARP inhibition may be a potential therapeutic approach in PD. 

Parkin is an E3 ubiquitin ligase involved in a variety of cellular processes. Transgenic expression in mice of a parkin substrate, aminoacyl TRNA synthetase complex interacting multifunctional protein 2 (AIMP2), results in progressive loss of dopaminergic neurons and motor impairment by activation of PARP-1, and hence paranthanos [260]. These deficits were rescued by administering a pharmacological inhibitor of PARP-1 (AG014699) [260]. AIMP2 is also required for the formation of α-synuclein aggregates and Lewy bodies [261].

PARP proteins contain two zinc-finger motifs, and thus they are targets for reactive nitrogen species (RNS) generated from NO [262]. Paranathanos induced by MPTP administration requires neuronal NO synthase [256], indicating that MPTP-induced PARP activation and subsequent polymerisation of ADP-ribose is associated with NO-induced DNA damage. Furthermore, NO levels are significantly elevated in nigral cells in PD [263,264]. In summary, these findings highlight parthanatos as a significant cell death mechanism associated with DNA damage in PD. 

#### 7.2.3. DNA Damage, p53, and Neuronal Death in PD

Similar to AD, there is accumulating evidence that p53 mediates the selective death of affected neurons in PD. Elevation of p53 has been detected in dopaminergic neurons derived from embryonic stem cells [210,265], and p53 mediates neurodegeneration in SN4741 cells, a mouse SNpc-derived cell line [266], and in in vivo [266] models of PD. Parkin also regulates p53 levels in the brain by transcriptional repression [267]. The neurotoxin 6-hydroxydopamine (6-OHDA) is commonly used to induce selective degeneration of dopaminergic and noradrenergic neurons, and thus model the symptoms of PD. The BH3-only protein PUMA, which is induced by p53, mediates death of primary dopaminergic neurons following 6-OHDA treatment by a mechanism involving DNA damage [268]. PUMA also mediates mitophagy in PD [269]. Interestingly, Parkin also inhibits 6-OHDA and mediates neuronal death via p53 [267]. Likewise, DJ-1, which is mutated in an autosomal recessive form of PD, inhibits the transcriptional activity of p53 [266]. Hence, these findings demonstrate that p53 plays a role in neuronal death in PD [270]. 

### 7.3. Amyotrophic Lateral Sclerosis (ALS)

ALS is a neurodegenerative disease characterised by progressive muscle weakness, fasciculations, spasticity, and eventual paralysis, due to death of both upper and lower motor neurons (MNs) in the brain, brainstem, and spinal cord [271]. Several mechanisms are implicated in the pathophysiology of ALS, including ER stress [272], mitochondrial dysfunction [273], oxidative stress [274], disruption in intracellular trafficking [275], proteostasis disruption [276], excitotoxicity [277], DNA damage [278], and abnormal RNA homeostasis [271,279]. The major hallmark of ALS is the formation of pathological forms of TAR-DNA binding protein 43 (TDP-43) in affected neurons and glia in 97% of cases [280]. This involves the abnormal aggregation and misfolding of TDP-43, hyperphosphorylation, truncation, and its mis-localisation from the nucleus to the cytoplasm [281]. TDP-43 performs important cellular functions in both DNA repair and RNA metabolism, and it bears striking functional and structural similarities to Fused in Sarcoma (FUS), and both proteins are mutated in familial ALS [278]. Interestingly, FUS is also found in a pathological form where it mis-localises to the cytoplasm in ALS [282]. Both loss of nuclear functions and gain of toxic functions in the cytoplasm of TDP-43 and FUS are implicated in pathophysiology. 

Mutations in approximately 50 genes are linked to ALS, including both TDP-43 and FUS, which together account for approximately 10% of familial cases [283]. The most frequent genetic causes of ALS are hexanucleotide repeat expansions (HREs) in the first intron of the chromosome 9 open reading frame 72 *(C9orf72)* gene, although this varies depending on the geographical population [284]. The C9ORF72 mutation encodes five dipeptide repeat proteins (DPRs) that are produced by non-canonical repeat associated translation [285]. Among the DPRs, proline–arginine (PR), glycine–arginine (GR), and glycine–alanine (GA) are thought to be the most toxic [285]. Mutations in superoxide dismutase 1 (SOD1) are the next most common cause of ALS [285]. Collectively, DNA damage is now strongly implicated in the pathophysiology of ALS, but the associated mechanisms of cell death remain unclear, similar to the other neurodegenerative conditions.

There is extensive evidence for DNA damage in ALS [1,149,286]. In addition, DNA damage has been detected at symptom onset in animal models of ALS [278], implying it may contribute to disease progression. Furthermore, there is a growing number of proteins associated with ALS (in both familial and sporadic cases) that possess physiological cellular functions in DNA repair, including TDP-43 and FUS, [278,287]. Several recent studies have shown that TDP-43 performs important functions in the DDR [278] in NHEJ DNA repair, where it is recruited to sites of DNA damage [278] with the XRCC4-DNA ligase IV complex [288]. Depletion of TDP-43 inhibits the formation of DNA damage foci in NSC-34 cells, suggesting that TDP-43 also functions in the recognition of DSBs [278]. In contrast, ALS-associated mutations in TDP-43 induce DNA damage and are associated with TDP-43 pathology in a mouse model [278]. Hence, these studies emphasise the fundamental role of TDP-43 in the maintenance of genomic integrity.

FUS displays similar functional properties as TDP-43 in both RNA metabolism and DNA repair [289]. FUS indirectly regulates HR and NHEJ in primary mouse neurons via binding to histone deacetylase 1 (HDAC1), and FUS recruitment to DSBs is necessary for efficient DDR signalling and DNA repair [290]. DNA damage is also present in motor neurons in FUS mouse models, including transgenic mice expressing mutant FUS^R521C^ [291]. DSBs are also known to induce FUS phosphorylation via DNA-PK and ATM [292,293]. 

Several studies have shown that C9orf72 repeat expansions induce genomic instability and neurodegeneration. DNA damage is triggered in C9orf72-ALS by the formation of abnormal nucleic acid structures, R loops, and G quadruplexes by the expanded repeat [294]. Dysfunction in DNA repair and DNA damage, indicated by 53BP1 and pATM repair foci, reduced H2A ubiquitylation, and more DSBs, are present in C9orf72 ALS patient spinal cord tissues [295,296]. Similarly, DNA damage was detected in iPSC motor neurons derived from C9ORF72-ALS/FTD patients displaying poly-GR aggregates [297]. DNA damage has been detected at symptom onset in ALS patients, implying it may contribute to disease progression.

Early studies first described mitochondrial DNA damage in relation to oxidative stress in ALS, but it is now apparent that many aspects of the DDR are perturbed in ALS. Previous studies examining spinal cords of ALS patients suggested that motor neurons have compromised mitochondrial function and oxidative DNA damage [298,299,300]. Furthermore, activity of the apurinic/apyrimidinic enzyme (APE1), one of the major participants of BER, is reduced in the frontal cortex of ALS patients [299]. However, another study demonstrated that the activity of APE1 is significantly increased in astrocytes in spinal cords in familial ALS, implying that BER is activated [301]. 

Mutations in the senataxin gene (*SETX*), which encodes a DNA/RNA helicase that functions in the DDR, are associated with juvenile forms of ALS (ALS4) [302]. In addition, mutations in the apurinic/apyrimidinic endodeoxyribonuclease-1 (*Apex1*) gene have been reported in both sALS and fALS tissues [303]. Upregulation of γH2AX in post-mortem spinal cords of C9ORF72 ALS patients and iPSC-derived motor neurons has also been detected [285,296]. Furthermore, increased γH2AX expression was detected in the spinal cord, frontal cortex, and striatum in the transgenic SOD1^G93A^ mouse model [304]. In cells expressing the FUS^R244C^ mutant, reduced amounts of pATM and histone deacetylase 1 (HDAC1) at DNA DSBs have been observed in U2OS cells, suggesting the presence of impaired DDR and DNA repair [290]. DNA damage and RNA splicing defects have also been detected in FUS^R521C^ transgenic mice [291].

Mutations in the never-in-mitosis A related protein kinase 1 (*NEK1*) gene, also involved in the DDR, have also been described in ALS [305]. Similarly, C21ORF2 stabilizes NEK1 and is required for efficient HR DNA repair, and it is also implicated as an ALS gene [306]. Furthermore, γH2AX expression was increased in NEK1-ALS iPSC-derived motor neurons compared to controls, suggesting the presence of DNA damage [305]. Decreased levels of NEK1, up to 50%, were also reported in NEK1-ALS patients [305]. In addition, elevated DNA damage has been demonstrated following NEK1 knockdown in Nek1 −/− murine cells [307]. Similarly, following DNA damage induction, *NEK1*-ALS motor neurons and NEK1 knockdown cells exhibited accumulation of γH2AX [308].

In ALS, several mechanisms have been identified by which the affected neurons die, including apoptosis, parthanatos, ferroptosis [309], and necrosis [310]. However, similar to AD and PD, only apoptosis and parthanatos have been described in relation to DNA damage. We describe this evidence below.

#### 7.3.1. DNA Damage and Apoptosis in ALS

There is extensive evidence that motor neurons die by apoptosis in ALS, and some of these studies have been linked to DNA damage. Several links between mitochondrial apoptosis and DNA damage have been described. Increased expression of OGG1was present in the nuclei of spinal cord motor neurons in SOD1^G93A^ mice [311]. In addition, increased oxidative DNA damage and decreased levels of Pol ɣ, were identified in this model [311,312,313]. Furthermore, decreased mitochondrial OGG1 activity was present in motor neurons of sALS subjects, linking impairment of mitochondrial DNA repair to the most common, sporadic forms of disease. Likewise, increased amounts of 8-hydroxy-2′-deoxyguanosine (8-OHdG), an indicator of oxidative DNA damage, and induction of BER, were observed in spinal cords of both sporadic and familial ALS patients, and in neuronal DNA from sporadic ALS subjects [298]. Degeneration of motor neurons was also present in a mouse model defective in the DNA repair protein ERCC1 (Δ/-), thus linking defects in DNA repair to degeneration of motor neurons [314]. 

Cytoplasmic TDP-43 expression in mice results in drastic motor neuron loss in the brain and the presence of typical phenotypic features of ALS. However, clearance of TDP-43 in the cytoplasm [315] reverses these phenotypes, implying that TDP-43 pathology is central to neurodegeneration in ALS. Pathological forms of TDP-43 contain N-terminal truncated C-terminal fragments that are deposited into inclusions, and these fragments are produced by the activity of caspases [316]. This implies that the activation of caspases during disease enhances TDP-43 pathology, and thus toxicity, by promoting TDP-43 cleavage. Notably, mis-localisation of TDP-43 in mouse neurons is enough to induce apoptosis, indicating that the cytoplasmic presence of TDP-43 is responsible for inducing neuronal death [317]. Notably, TDP-43 induces cell death by upregulation of Bim expression and downregulation of Bcl-xL [316]. Expression of cyclin-like protein Spy1, which inhibits DNA-damage-induced apoptosis, is decreased in SOD1^G93A^ overexpressing cells, as well as in the spinal cord of SOD1^G93A^ transgenic mice [318]. Decreased levels of Spy1 therefore may inhibit the DDR and enhance DNA damage and apoptosis. 

DNA damage was detected with the presence of apoptosis markers cleaved PARP1 and cleaved caspase-3 in TDP-43 CRISPR/Cas9 knockout SH-SY5Y cells [319]. Similarly, progressive elevation in apoptosis after depletion of TDP-43 was detected in TDP-43 CRISPR/Cas9 knockout SH-SY5Y cells [319]. Together, these findings suggest that loss of TDP-43 is strongly associated with accumulation of unrepaired DSB, persistent DDR activation, and consequently apoptotic cell death in neurons. Furthermore, TDP-43 performs multiple functions in RNA metabolism, including transcriptional regulation, RNA splicing, microRNA biogenesis, mRNA stability, and translation [320]. Mutant TDP-43 leads to extensive RNA splicing defects [321], including those associated with the apoptotic and mitotic cell death pathways [321], further linking apoptosis to TDP-43 in ALS. 

Nucleophosmin (NPM1, also known as B23) is an abundant nucleolar protein that regulates both DNA repair and apoptosis [322] by regulating the levels of APE1 and hence BER [322]. In C9orf72 patient tissues, NPM1 interacts more with APE1 than in control patients [296], and overexpression of NPM1 inhibits apoptosis in cells expressing polyGR and polyPR [296]. These data thus link defects in the BER pathway with apoptosis in C9orf72-ALS. Furthermore, the C9orf72 DPR polyPR inhibits the function of NPM1 in NHEJ and homology-directed DSB DNA repair pathways, also linking these mechanisms to apoptosis in C9orf72-ALS [285]. Several other studies have demonstrated that apoptosis of motor neurons is induced by the C9orf72 mutation, although this was not examined in relation to DNA damage [323].

Similarly, induction of DNA damage by mutant SOD1 has also been linked to apoptosis. DNA damage, increased p53 activity, and more apoptosis were observed in SH-SY5Y cells expressing mutant SOD1^G93A^ [324]. Unusually, mutant SOD1 localised in the nucleus rather than the cytoplasm, where it was associated with DNA. These results indicate that mutant SOD1 induces DNA damage and triggers apoptosis by activating p53 [324]. Motor neuron degeneration was also linked with accumulation of both DSBs and SSBs in mutant SOD1^G93A^ transgenic mice [325]. 

Emerging evidence has suggested that the DNA repair capacity of neurons can be enhanced by upregulation of brain- derived neurotrophic factor (BDNF) [326]. BDNF was identified as a target of FUS^R521C^–associated DNA oxidative damage and RNA splicing defects in mice [291]. Mutant FUS^R521C^ formed a more stable complex with BDNF RNA, which contributed to BDNF splicing defects and impaired BDNF signalling through receptor tropomyosin receptor kinase B (TrkB) [291]. The anti-apoptotic properties of BDNF have been described previously [327,328]. Trophic factor deficiency stimulates neuronal NO synthase and apoptosis in rat embryonic motor neurons in vitro [329]. In the presence of BDNF, NO is essential for the survival of motor neurons [329]. However, in the absence of BDNF, by forming peroxynitrite [329], the neurogenesis properties of NO result from increasing cyclic guanosine 5′ monophosphate (cGMP) production [328]. Activation of apoptosis was detected following expression of neuronal NO in the absence of trophic factors in cultured motor neurons. Interestingly, in rat motor neurons deprived of BDNF, cGMP inhibits neuronal NO-induced apoptosis [328]. Moreover, BDNF was protective against 3-nitropropionic acid (3-NP)-induced activation of apoptotic proteins from mitochondria, and activation of caspase-3 in primary rat cortical neurons.

RNF168-induced H2A ubiquitination regulates 53BP1 and ATM-mediated repair [330]. p62 downregulation or overexpression of RNF168 improved DSB repair and decreased levels of DSBs in cells overexpressing C9orf72 mutations, indicating that defective autophagy, induced by p62 accumulation, is a major cause of genome instability in C9orf72-ALS disease models [295]. Furthermore, expression of C9orf72 DPRs halted ATM signalling in MRC5 cells [295]. In addition, increased levels of TOP1cc and neuronal cell death (by an undefined mechanism) were observed following ATM deficiency in primary neural cultures and rodent models [331]. ATM activation inhibits apoptosis by inducing insulin-mediated pathways in the differentiated human neuron-like SH-SY5Y cells [332]. 

#### 7.3.2. DNA Damage and Parthanatos in ALS

Similar to AD and PD, there is also evidence that DNA damage induces parthanatos in ALS. Enhanced PARP1 expression was detected in the motor cortex, parietal cortex, and cerebellum of ALS brains, and was localised with TDP-43 inclusions [333,334]. In addition, the PARP-1 inhibitor veliparib diminished PAR levels in the nuclei of motor neurons in spinal cords of ALS patients and inhibited the formation of cytoplasmic TDP-43 aggregates in those neurons [335]. PAR signalling to mitochondrial AIF is a major event in the initiation of parthanatos, and translocation of AIF release from mitochondria into the nucleus was detected in spinal cords of mutant SOD1^G93A^ mice, indicating a role for AIF in initiating motor neuronal death in ALS [336]. Upregulation of PARP-1 was also detected in the motor cortex, parietal cortex, cerebellum, and astrocytes of spinal cords of sALS patients, although it was downregulated in spinal cord motor neurons [313,333,337]. FUS overexpression in the nucleus induces apoptosis of motor-neuron-like NSC34 cells and PARP cleavage [338]. 

TDP-43 and FUS mis-localisation from the nucleus to the cytoplasm has been implicated in the inhibition of DNA repair in ALS. Notably, the RGG domain of FUS is capable of binding to PAR, where it localizes at sites of DNA damage [339]. Conceivably, mutations this domain may therefore have a greater capability to dysregulate PAR in the repair of damaged DNA.

#### 7.3.3. DNA Damage, p53, and Neuronal Death in ALS

Similar to the other neurodegenerative conditions, p53 is implicated in neuronal death in ALS. Several of the studies already described above demonstrate a relationship between p53 and DNA damage in ALS. In addition, the levels of p53 are aberrantly increased in the motor cortex and spinal cord of ALS patients [340]. Furthermore, overexpression of mutant SOD1^G93A^ induces DNA damage and apoptosis through activation of p53 in SH-SY5Y human neuroblastoma cells [324]. In addition, recently p53 was implicated as a major player regulating the death of iPSC motor neurons expressing C9orf72 polyPR [341]. Decreased levels of p53 in these cells also ameliorated axonal degeneration mediated by polyGA, and improved survival in a C9orf72 fly model [341]. However, a reduction in PUMA levels in mouse cortical neurons prevented polyPR_50_ toxicity, and PUMA was a downstream effector of p53-associated cell death [341]. Activation of p53 in mutant SOD1^G86R^ mice was detected with apoptosis by modulating of the Bcl-x/Bax ratio [342].

### 7.4. Frontotemporal Dementia (FTD)

Frontotemporal dementia (FTD) is a progressive neurodegenerative disorder that mainly affects the frontal and anterior temporal cortex, leading to impairment of executive functioning, behavioural changes, and a decline in language proficiency [343]. FTD is the second most common form of dementia in individuals younger than 65 years [343], and ALS and FTD are thought to form opposite ends of the same disease spectrum [344]. Several mechanisms are implicated in the pathophysiology of FTD, including ER stress [345], mitochondrial dysfunction [346], oxidative stress [347], disruption in intracellular trafficking [275], proteostasis disruption [320], excitotoxicity [348], DNA damage [347], and abnormal RNA homeostasis [320]. ALS and FTD also have significant pathological and genetic overlap [349]. Several mechanisms have been implicated in neuronal death in FTD, including apoptosis [350], necrosis, parthanatos, and ferroptosis [351]. 

DNA Damage and Apoptosis in FTD 

In the superior frontal gyrus and anterior pole of temporal lobes obtained from FTD patients, increased activation of caspase-3 was identified compared to controls, implying induction of apoptosis [350]. Increased DNA damage was also identified in degenerating astrocytes, indicating that DNA damage and apoptosis are related in glia in FTD [350]. 

TDP-43 pathology is present in FTD patients with mutations in the gene encoding progranulin (*GRN*), which causes 50% of cases [352,353]. Overexpression of *GRN* in glioblastoma cells was protective against tomozolomide-induced DNA damage [354]. Interestingly, lack of the progranulin homolog *pgrn-1* in *C. elegans* leads to enhanced clearance of apoptotic cells [355] and impairment in motor neuron development in zebrafish [356]. Progranulin binds to sortilin at the cell surface, which mediates apoptosis in response to neurotrophins [357]. 

### 7.5. Huntington’s Disease (HD)

Huntington’s disease (HD) is a progressive autosomal dominant disorder characterised by specific degeneration of enkephalin-containing medium spiny neurons in the basal ganglia [358], although the SNpc and cortex are also affected. It is caused by expansion of trinucleotide repeats (CAG) at the 5′-end of the gene encoding huntingtin (HTT) above a specific threshold [359]. The normal population contains 5–36 CAG repeats, whereas 38 or more repeats are found in HD, and disease severity is proportional to the number of repeats [360]. Several mechanisms are implicated in the pathophysiology of HD, including ER stress [361], mitochondrial dysfunction [362], oxidative stress [363], disruption in intracellular trafficking [364], proteostasis disruption [361], excitotoxicity [365], and DNA damage [366].

A role for DNA damage in the pathogenesis of HD has been reported [358,366,367], although its link to specific cell death mechanisms has not yet been described [368]. MSH2 is an important protein involved in many different forms of DNA repair, including mismatch repair, transcription-coupled repair [5], HR [6], and BER [7]. Studies of transgenic mice bearing exon 1 of human HD crossed with *Msh2*–/– have revealed that MSH2 is involved in somatic expansions of the polyQ tract [369,370]. This involves BER factors OGG1 and Nei-like DNA glycosylase (NEIL1) [371]. Interestingly, a genome-wide association study of 6000 to 9000 genetically diverse HD patients identified genes involved in DNA damage repair as the major modulators of HD in terms of severity and age of disease onset [366]. Enhanced OH(8)dG staining in tissues from animals at late-stage disease in a transgenic mouse model of HD^R6/2^ indicated that oxidative DNA damage may play a role in the progression of neurodegeneration in this model [365].

Several mechanisms of cell death have been described in HD pathogenesis, particularly apoptosis [372,373] and autophagic cell death [374]. In addition, a novel form of necrosis of neurons—transcriptional repression-induced atypical cell death (TRIAD)—was recently reported in HD [375]. However, none of these mechanisms have been associated with DNA damage, and thus are not detailed here.

DNA Damage, p53, and Neuronal Death in HD

There are several lines of evidence that p53 is associated with neuronal death in HD. Increased levels of p53 are present in the brains of HD patients, as well as mHtt^N171−82Q^ transgenic (mHtt-Tg) mouse models [376]. Induction of p53 and decreased basal levels of Bcl-2 have been suggested as a major contributor to the selective vulnerability of striatal medium spiny neurons to degeneration [377]. In addition, the interaction of mutant huntingtin and p53 inclusions has been detected both biochemically [378].. Activation of p53 and increased phosphorylation of H2AX and ATM were reported in rat pheochromocytoma PC12 cells expressing mutant Htt14^A2.6^ [379]. Interestingly, induction of DNA damage was detected prior to mutant huntingtin aggregate formation and HD pathogenesis [379]. Hence, these data imply that p53 is an important factor in neuronal death in HD, similar to the other neurodegenerative diseases described above.

## 8. Therapeutic Potential of DNA Damage in Preventing Cell Death in Neurodegenerative Diseases

As detailed above, preserving genomic integrity is critically important in neurons, given their unique characteristics. If DNA is unrepaired or repaired incorrectly, DNA damage results, which can trigger diverse types of neuronal death in neurodegenerative disorders. However, there are currently no effective treatments that can prevent the death, and hence loss, of specific types of neurons in these conditions. Therefore, understanding how DNA damage induces neurodegeneration and each form of cell death may allow for the development of therapies for neurodegenerative diseases in the future. Various DNA repair processes, such as those mediated by ATM [380], BRCA1 [381,382], Ku80 [383], DNA-PKc [384], PARP1 [385], HDAC1 [168], and GSK3β [386], correspond to potential targets that could be utilised for therapeutic strategies. However, these mechanisms have not been examined in preclinical models of neurodegenerative conditions or in clinical studies. Similarly, markers of neuronal genome instability may also have potential as prognostic or diagnostic biomarkers for these diseases, but this has been relatively unexplored. Antisense oligonucleotides (ASOs) targeting specific mutations in neurodegenerative conditions have also been showing promise, although they have pharmacological limitations. Nevertheless, small molecules or ASOs that target the DDR or enhance DNA repair may have the ability to prevent specific types of neuronal death, and thus therapeutic potential for these conditions.

## 9. Conclusions

DNA damage is now well described in neurodegenerative diseases, and it is relatively straightforward to envisage how this process triggers neuronal death. The DDR initially aims to maintain survival of the cell, but it also has the capacity to actively induce death when DNA damage becomes prolonged or severe. The number of newly characterised cell death pathways has been increasing in recent years, and several of these are linked to DNA damage. Similarly, an increasing number of DNA repair pathways are perturbed in neurodegeneration, highlighting this as an important disease process. However, whilst multiple cell death mechanisms have been linked to DNA damage, surprisingly few have been associated with these diseases. Nevertheless, DNA repair pathways intersect significantly, so it can be difficult to discern experimentally which pathways induce specific forms of cell death. It is also important to note that cell death induced by defective DNA repair may also be passive, by interfering with replication, transcription, or functioning of DNA, as well as by specific regulated cell death responses. Moreover, many cell death mechanisms described in other cell types have not yet been detected in neurons. In this review, we aimed to summarise the current understanding of this new field.

**Figure 3:**
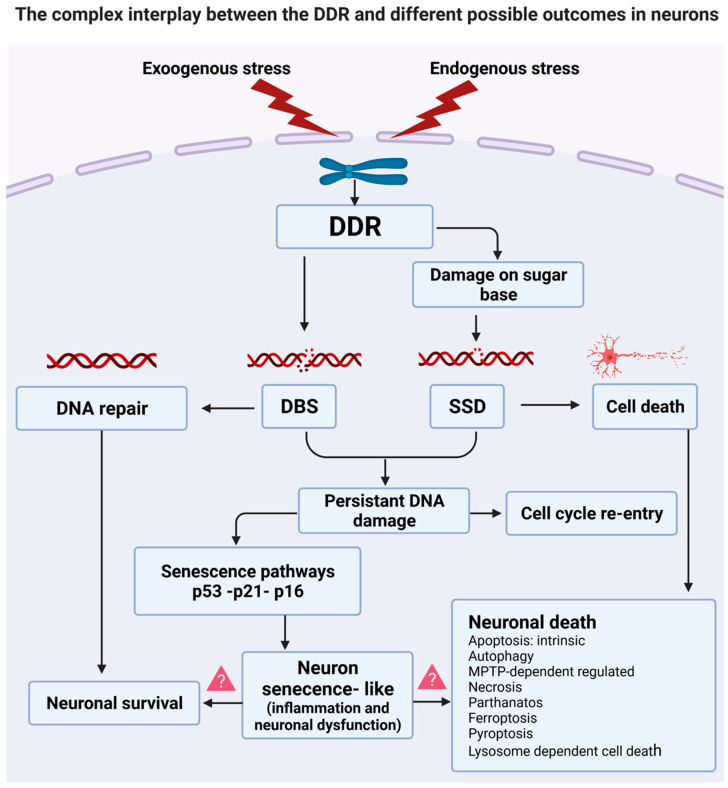
The complex interplay between the DDR and different outcomes in neurons. Neurons are exposed to both endogenous and environmental/exogenous sources of DNA damage. The DDR recognizes the damage and activates downstream factors, resulting in distinct outcomes depending on the damage. This includes several key proteins that contribute through diverse pathways to mediate either survival or RCD. The figure depicts those RCD pathways induced by DNA damage amongst the numerous cell death pathways now identified. As the DDR requires intervention of multiple pathways to maintain the integrity of the genome, the interplay between different cell death pathways is complex and difficult to discern. Thus, a deeper understanding of how the DDR induces neuronal death may lead to new therapeutic opportunities in neurodegenerative diseases to prevent or delay the loss of neurons [387,388].

DNA repair mechanisms decline significantly during aging, and the biggest risk factor for neurodegenerative diseases is advancing age. Hence, these conditions are likely to increase greatly in the coming decades due to the aging population. Given that the aberrant death of neurons is the major pathological feature of these diseases, it is important to fully define these mechanisms so that therapeutic strategies can be designed to prevent their loss in the future. In contrast, cell death mechanisms relevant to the DDR have been explored in detail in relation to cancer where agents that induce DNA damage are used as therapeutics. However, the links between DNA damage and cell death have been relatively unexplored in neurodegenerative diseases. Hence, additional research in this area is important to develop a deeper understanding of these events. Figure 3 schematically illustrates the interplay between the DDR and cell death outcomes in neurons. More detailed knowledge of how DNA repair and the DDR can be used therapeutically to prevent or delay neuronal loss in these conditions is therefore required in the future.

## Figures and Tables

**Table 1:** Classification of regulated cell death (RCD) mechanisms. Cells exposed to perturbations of the intracellular/extracellular environment activate various signalling pathways that lead to distinct types of RCD. These types are categorised by the NCDD based on their molecular and cellular features.

Pathway	Form of Apoptosis?	NCDD Definition	Intra- or Extracellular Trigger?	Described in Neurons?	Related to DNA Damage in Neurons?
Apoptosis Intrinsic	Yes	Demarcated by MOMP (both) or CASP8 (extrinsic) and induced by executioner caspases, mainly CASP3	Both	Yes	Yes
Apoptosis Extrinsic	Yes	Demarcated by MOMP (both) or CASP8 (extrinsic) and induced by executioner caspases, mainly CASP3	Extracellular	Yes	No
Anoikis	Yes	Form of intrinsic apoptosis elicited by loss of integrin-dependent attachment to extracellular matrix	Both	No	No
MPT-driven necrosis	Non-apoptotic	Initiated by oxidative stress or Ca2+ overload, necrotic phenotype, dependent on CYPD	Intracellular	Yes	Neurotoxic
Necroptosis	Non-apoptotic	Depends on MLKL, RIPK3, and sometimes RIPK1 activity	Both	Yes	No
TRIAD	Non-apoptotic	Transcriptional repression-induced atypical cell death	-	Yes	No
Ferroptosis	Non-apoptotic	Initiated by oxidative stress, depends on GPX4	Intracellular	Yes	Yes
Pyroptosis	Non-apoptotic	Depends on plasma membrane pores by gasdermin proteins, inflammatory caspases	Both	Yes	Yes
Parthanatos	Non-apoptotic	Initiated by PARP1 hyperactivation, coupled to AIF and MIF DNA degradation	Intracellular?	Yes	Yes
Entosis	Non-apoptotic	Initiated from actomyosin-dependent internalisation executed by lysosomes	Intracellular?	No	No
NETotic cell death	Nonapoptotic	ROS-dependent restricted to hematopoietic cells, associated with NET extrusion		No	No
Lysosome-dependent cell death	Non-apoptotic	Demarcated by primary LMP and triggered by cathepsins or MOMP and executioner caspases	Intracellular?	Yes	No
Autophagy-dependent cell death	Non-apoptotic	Depends on the autophagy machinery	Intracellular?	Yes	No
Immunogenic cell death	Non-apoptotic	Activates an adaptive immune response in immunocompetent hosts		No	No
Oxeiptosis *	Non-apoptotic	Activated by ROS, dependent on KEAP1, mediated by PGAM5 and AIFM	Intracellular?	No	No
Alkaliptosis *	Non-apoptotic	Activated by pH changes, an alkalinisation-dependent form of regulated necrosis.	Intracellular?	No	No

Table Abbreviations: MOMP: outer mitochondrial membrane permeabilisation; ROS: reactive oxygen species; MPTP: mitochondrial permeability transition pore; KEAP1: Kelch-like ECH-associated protein 1; CASP: caspase I; PGAM5: phosphoglycerate mutase 5; AIFM: apoptosis-inducing factor mitochondria associated; LMP: latent membrane protein; NET: neutrophil extracellular traps; CYPD: cyclophilin D; MLKL: mixed-lineage kinase domain-like pseudokinase; RIPK3: receptor-interacting serine/threonine kinase 3; GPX4: glutathione peroxidase 4. * Not yet described in the NDCC guidelines.

## Data Availability

Not applicable.
